# Evaluating Sepsis Mortality Predictions from the Emergency Department: A Retrospective Cohort Study Comparing qSOFA, the National Early Warning Score, and the International Early Warning Score

**DOI:** 10.3390/jcm14144869

**Published:** 2025-07-09

**Authors:** German Alberto Devia-Jaramillo, Lilia Erazo-Guerrero, Vivian Laguado-Castro, Juan Manuel Alfonso-Parada

**Affiliations:** 1 Department of Emergency Medicine, Hospital Universitario Fundación Santafé de Bogotá, Bogotá 110111, Colombia; lilyerazo@gmail.com (L.E.-G.); vivilaguado@hotmail.com (V.L.-C.); 2 School of Medicine, Universidad del Rosario, Bogota 110111, Colombia; juanm.alfonso@urosario.edu.co

**Keywords:** NEWS, IEWS, SOFA, Sepsis, diagnosis, mortality, qSOFA

## Abstract

**Introduction**: Sepsis has a high mortality rate, especially in low-income countries. Improving outcomes depends on the early recognition of patients at risk of death. Therefore, rapid and applicable prediction scores are needed in emergency triage. **Objective**: This study assessed the effectiveness of the qSOFA, NEWS, and IEWS scales in predicting in-hospital mortality during emergency triage. Additionally, we analyzed the efficacy of the IEWS_L, which integrates the IEWS with arterial lactate levels measured upon admission to the emergency department. **Method**: This retrospective study included patients who consulted the emergency department with suspected sepsis, where various scoring systems were evaluated for their effectiveness. We evaluated the diagnostic capacity of the tests by measuring the specificity, sensitivity, positive and negative predictive values, as well as the areas under the curve (AUC) of each score to predict mortality. **Results**: The study included 383 patients who had visited the emergency department. The overall mortality rate was 20.6%, and the mortality rate, precisely due to septic shock, was 35.2%. The AUC values for predicting in-hospital deaths due to sepsis were as follows: qSOFA: 0.68 (95% CI: 0.62–0.74); NEWS: 0.71 (95% CI: 0.64–0.77); IEWS: 0.74 (95% CI: 0.68–0.80); IEWS_L: 0.81 (95% CI: 0.76–0.86). **Conclusions**: In the emergency department, the IEWS scale demonstrated the best ability to accurately predict in-hospital mortality from sepsis when compared to the qSOFA and NEWS scale. Additionally, incorporating the serum lactate level into the IEWS scale significantly enhances its capacity to predict mortality.

## 1. Introduction

Sepsis is a disease with high mortality in the world [1], especially in middle- to low-income countries such as those in Latin America [2]. In some middle- to low-income countries, mortality rates from sepsis are 21.8%, and from septic shock, they range from 45.6% to 56.4% [3,4]. Despite advances in different sepsis therapies, since 2001, no treatment protocols have been demonstrated that show a significant reduction in mortality from sepsis worldwide [5]. This high mortality rate results not only from ineffective treatments but also from a lack of consensus in sepsis diagnosis [6]. Despite existing management guidelines, diagnosing sepsis remains challenging due to a lack of adequate, precise, and user-friendly diagnostic tools [7].

Overcrowding in emergency services leads to more extended stays, and this delay is associated with worsening clinical outcomes [8]. Since sepsis is a disease that depends on the timing of interventions [9], recognizing sepsis early is crucial for reducing mortality rates. Therefore, it is essential to implement rapid detection strategies that can be used in the triage room. These strategies will help emergency department staff identify patients with suspected sepsis who are at increased risk of death. By doing so, timely interventions can be initiated, ultimately leading to improved clinical outcomes.

Sepsis management guidelines recommend using scales such as the Quick Sequential Organ Failure Assessment (qSOFA) [10]. However, there are reports of the low predictive capacity of this scale for death, with an AUC of 0.59 (95% CI 0.58–0.60) [11,12]. For this reason, it should not be used as the only prediction tool [10]. Because of this low capacity, the National Early Warning Score (NEWS) has become a widely used scale for predicting death from sepsis in the emergency room [13]. The scale was created in the United Kingdom using retrospective patient data, and its ability to distinguish patients has been successfully validated, including through several meta-analyses [14,15]. However, in populations from low- to middle-resource settings, the predictive ability shows diverse results, with an AUC of 0.68 (95% CI: 0.55–0.82) [16] to 0.78 (95% CI:0.72–0.83) [17]. The International Early Warning Score (IEWS) [18] adds other population parameters. It has been shown to improve the ability to predict death from sepsis in the emergency room compared to the NEWS [18]. The scale offers the advantage of incorporating parameters such as patient age and sex, enabling a more personalized assessment than scales that rely solely on vital signs. These scales are designed to predict mortality from sepsis but not to diagnose this disease. This study aimed to evaluate the discriminatory capacity of the qSOFA, NEWS, and IEWS scales in predicting in-hospital mortality when applied in the triage room of the emergency department. Additionally, the study aimed to assess the discriminatory capacity of the IEWS_L scale for mortality; the scale is defined as the sum of the IEWS scale value and the arterial lactate level measured upon admission to the emergency department.

## 2. Methods

### 2.1. Study Design

This retrospective study aimed to validate the effectiveness of the Early Warning Score (EWS) and the quick Sequential Organ Failure Assessment (qSOFA) scales in predicting in-hospital mortality due to sepsis. It also aimed to evaluate the discriminatory ability of a new scale developed specifically for this research: the IEWS_L. This study collected data from patients registered in the Institute of Urgencies and Trauma (ISMET) sepsis database at the Hospital Universitario Fundación Santa Fe de Bogotá, Colombia, from June 2023 to December 2024.

### 2.2. Eligibility Criteria

The study was conducted in a fourth-level university hospital in Bogotá, Colombia. The inclusion criteria were adult patients over 18 years of age evaluated in the triage area with suspected sepsis, regardless of its origin, according to the Sepsis-3 consensus [10]. Patients admitted from other institutions and pregnant patients were not included. Additionally, patients without a confirmed infection were also excluded. All eligible patients were included sequentially until the desired sample size was achieved.

### 2.3. Methodology

#### 2.3.1. Definition of Infection

The diagnosis of infection was confirmed if the patient meets at least one of the following criteria: (i) a positive blood culture that shows a non-colonizing or non-contaminating agent; (ii) a positive urine culture (more than 100,000 CFU/mL), accompanied by urinary symptoms or sepsis without another apparent source; (iii) a positive culture from an endobronchial aspirate, sputum, or bronchoalveolar lavage; (iv) a positive culture from ascitic fluid (>250 polymorphonuclear cells per field); (v) a positive stool culture; (vi) or imaging evidence of intra-abdominal collections or pulmonary consolidation in the absence of the above.

#### 2.3.2. Definition of Sepsis

Sepsis was diagnosed according to the current definition of sepsis [10]. A case was classified as sepsis when both an infection was confirmed, and evidence of organ dysfunction was indicated by a Sequential Organ Failure Assessment (SOFA) score of 2 or more points [10].

### 2.4. Sample Size

An anticipated mortality rate of 18% was established, with a significant level of 95% and a power of 80%. We identified a relevant difference of 0.1 between the areas under the curve (delta), using an AUC of 0.6 as the reference benchmark. Based on these parameters, we confidently determined a minimum sample size of 375 patients to include in the study. This calculation used the *pwr* and *pROC* packages in RStudio, version 2024.12.1 + 563, Copyright (C) 2025, by Posit Software.

### 2.5. Data Analysis

Initially, the data recorded in the database used were reviewed to avoid inconsistencies or duplications, ensuring the accuracy of the data and each variable. A descriptive analysis of the study variables was subsequently performed. For categorical variables, the frequency distribution was used. Regarding continuous variables, central tendency measures were calculated according to the distribution type. Normal distribution was expressed as means and standard deviations, and medians and interquartile ranges (IQRs) were reported for non-normal distributions. The Shapiro–Wilk test for normality was used. The sensitivity, specificity, and area under the curve (AUC) were documented using standard formulas for binomial proportions and corresponding 95% confidence intervals (CI). Adjustments were made to analyze positive predictive value (PPV) and negative predictive value (NPV) according to the prevalence of the disease found in the study. The association between categorical variables and mortality was established by calculating the relative risk and checking independence using the chi-square test. We used the DeLong test from the pROC package in R to demonstrate differences between ROC curves. A *p*-value of less than 0.05 was considered statistically significant. We evaluated the calibration performance of the qSOFA, NEWS, IEWS, and IEWS_L scales. We applied a bootstrap method with 1000 replicates for each test. We used the bootstrap method for the calibration curve because the data were not normally distributed, the sample was not very large, and we considered that this method could mitigate some potential biases. All statistical calculations were performed using RStudio software, version 2024.12.1 + 563, Copyright (C) 2025, by Posit Software.

The study was conducted at the Hospital Universitario Fundación Santa Fe de Bogotá, Colombia, and was approved by the institution’s research and ethics committee with approval number CCEI-15338–2023.

## 3. Results

**Figure 1:**
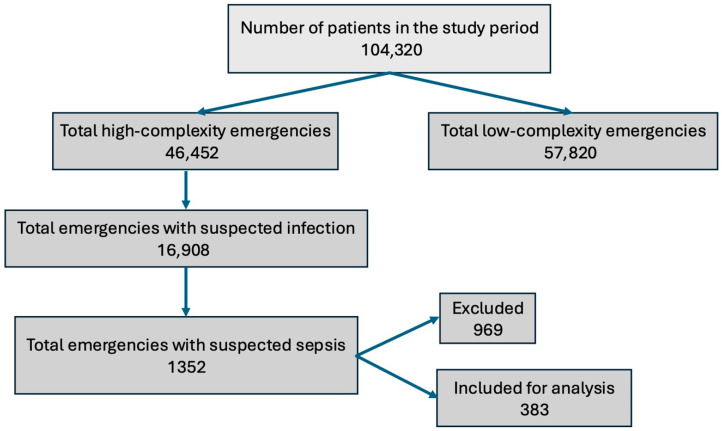
Flowchart for patient inclusion.

Patients initially considered were those whose reason for consultation was a suspicion of being an infectious process upon arrival to the emergency department. We reviewed this group of patients and discarded those whose medical evaluation determined that the reason for their consultation was not an infection but rather another explanation. Of the remaining group of patients, that is, those with infection, we determined which had sepsis and which did not using the SOFA score. The assessment was performed based on laboratory values, not at triage but in the emergency department. We excluded those who did not have a SOFA score of two or more points. The analysis included 383 patients (Figure 1), 969 patients were excluded for the following reasons: 98 were admitted to the emergency department from another institution, 652 did not meet Sepsis-3 criteria, 86 patients were pregnant, 92 patients could not be confirmed as infected, and 41 patients had incomplete data. Mortality was 20.6% (79 patients). Of the total patients, 156 (40.7%) presented septic shock. Mortality due to septic shock was 35.2%, while death in the sepsis-only group was 10.5%. No differences in mortality were observed between men and women. The median age of the study cohort was 73 years, and cardiovascular disease was the most common comorbidity (47.8%). A history of chronic kidney disease, cardiovascular disease, and COPD was found to be significantly associated with mortality in the study group (Table 1). Furthermore, a significantly increased mortality was observed in patients with septic shock, with an odds ratio of 4.57 (95% CI 2.70–7.94) (Table 1). The most common site of infection was the lung, and no differences were found between the site of sepsis and mortality (Table 1). A significant difference was found in the values of all the scores evaluated (qSOFA, NEWS, IEWS, IEWS_L) and documented in-hospital mortality (Table 1).

**Figure 2:**
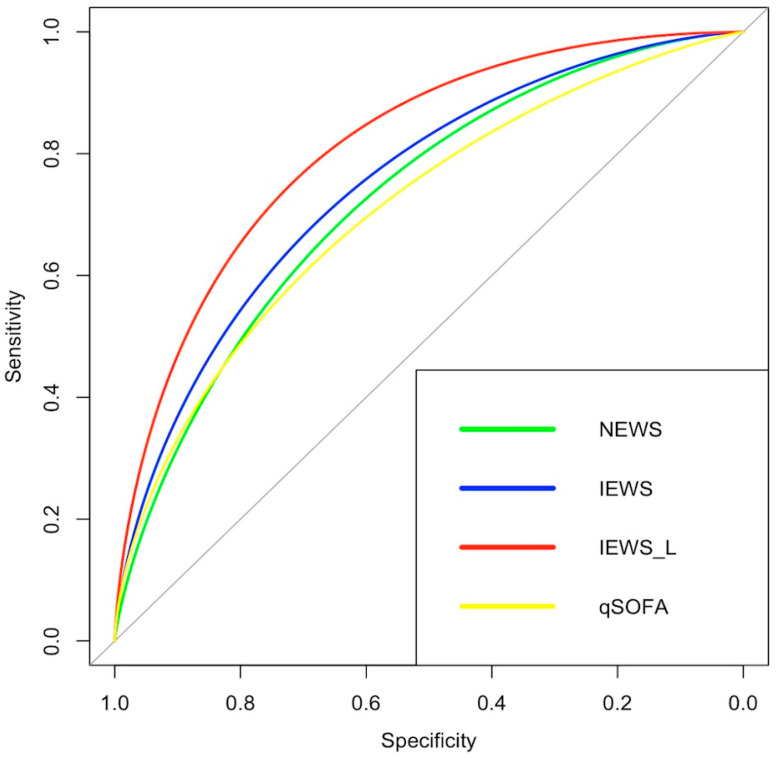
**Receiver operating characteristic curves comparing prediction tools**. qSOFA 0.68 (95% CI: 0.62–0.74); NEWS: 0.71 (95% CI:0.64–0.77); IEWS: 0.74 (95% CI:0.68–0.80); IEWS_L: 0.81 (95% CI:0.76–0.86).

Regarding the discriminatory capacity of the scales evaluated and their comparison, the highest ROC curve (AUC) value was found for IEWS, at 0.74 (95% CI: 0.68–0.80), while the lowest was found for qSOFA, at 0.68 (95% CI: 0.62–0.74). The AUC value for the NEWS scale was 0.71 (95% CI: 0.64–0.77). When comparing the AUC values of the scales using DeLong’s test, the IEWS value was significantly higher than that for the NEWS (*p* = 0.036) and qSOFA (*p* = 0.039) scales. However, although the AUC of the NEWS scale was higher than that of the qSOFA, 0.71 (95% CI: 0.64–0.77) and 0.68 (95% CI: 0.62–0.74), respectively, this difference was not statistically significant in the cohort (*p* = 0.293) (Figure 2).

**Figure 3:**
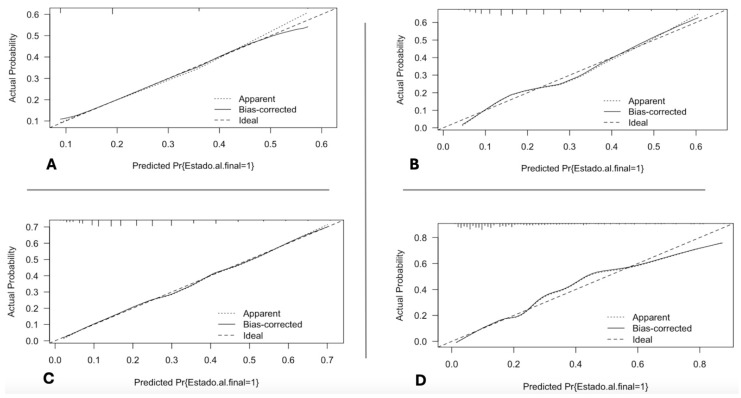
Calibration curves of prediction scales: (**A**) qSOFA, (**B**) NEWS, (**C**) IEWS, (**D**) IEWS_L.

Adding the lactate value to the original IEWS (IEWS_L) improved the scale’s AUC, 0.81 (95% CI: 0.76–0.86). The addition of lactate was performed by adding the total value of the IEWS scale plus the total value of the lactate obtained. Comparing the AUC of the IEWS_L scale with that of the other scales revealed significant differences: IEWS_L vs. IEWS *p* = < 0.001, IEWS_L vs. NEWS *p* = < 0.001, and IEWS_L vs. qSOFA *p* = < 0.001. We decided to add the lactate value to the total calculated value of the NEWS scale (NEWS_L), and we documented that the discrimination capacity is high (AUC: 0.794 (95% CI: 0.73–0.84)): when compared with the AUC value of the NEWS scale, a significant difference was documented *p* < 0.001. Likewise, we decided to compare the discrimination capacity of the NEWS_L and IEWS_L scales, it was found that although the AUC of IEWS_L is higher than NEWS_L, this difference is not statistically significant. *p* = 0.088. Calibration curves were created for all scores evaluated, and the original scores were found to behave appropriately. However, the calibration curve for adding lactate to the scores presents some problems, especially at high-scale values (Figure 3). The scale with the highest positive and negative predictive values was IEWS_L, although the scale with the highest specificity was qSOFA, and the scale with the highest sensitivity was IEWS_L (Table 2).

## 4. Discussion

Early detection and prediction of mortality from sepsis, particularly in the emergency department, is a current priority in clinical research [19]. However, emergency room care has particularities, such as short attention times due to the constant problem of overcrowding [20]. There is a need for straightforward death prediction tools that can be used in the triage room of the emergency department. The objective of this study was to evaluate the effectiveness of various scales that nurses can easily apply during triage. These tools aim to identify patients at high risk of death from sepsis early on, allowing for more timely interventions and ultimately improving clinical outcomes.

Regarding the predictive capacity of the scales evaluated in this study, the scale with the lowest predictive capacity was qSOFA with an AUC of 0.68 (95% CI: 0.62–0.74); the value is very similar to that reported in the meta-analysis by Wang et al. [14], which included 62,338 patients and demonstrated a predictive ability of qSOFA for mortality, with an AUC of 0.69 (95% CI: 0.65–0.73) [14]. The cohort of Abdullah et al. with 2045 patients demonstrated an AUC of 0.63 (95% CI: 0.58–0.67) for predicting mortality from the qSOFA scale [21]. Also, the study with 260 patients showed a low predictive capacity for mortality with this score in an emergency department, with an AUC of 0.605 (95% CI: 0.50–0.70) [22]. These data, taken together, concluded that the qSOFA score is not the best tool for predicting death in the emergency department. For this reason, the NEWS is routinely used in emergency departments. In our study, the predictive capacity of the NEWS was 0.71 (95% CI: 0.64–0.77); this value is similar to that of studies such as the meta-analysis by Wang et al., which reported a predictive capacity with an AUC of 0.69 (95% CI: 0.65–0.73) [14].

Being slightly lower than that of other studies reported with smaller populations, e.g., AUC of 0.78 (95% CI: 0.72–0.83) [17] and AUC of 0.77 (95% CI: 0.75, 0.80) [23], we assessed the discriminatory capacity of the qSOFA and NEWS scales. Although a better AUC was found for the NEWS scale, it was not significantly better than the qSOFA scale in the cohort studied. For this reason, better prediction tools are required that include other specific characteristics of the patients, making them more individualized to improve the prediction of clinical outcomes.

The IEWS scale is a variation of the NEWS scale. The scale includes, in addition to the parameters included in the NEWS scale, the age and sex of the patients. In a recent study conducted in the Netherlands and Denmark [18], the IEWS scale demonstrated a greater discriminatory capacity compared to the NEWS scale. In our cohort, we found that the predictive capacity of the scale was good (AUC of 0.74 (95% CI: 0.68–0.80)), albeit slightly lower than the discrimination capacity reported by Candel et al. [18] (AUC of 0.87 (95% CI: 0.85–0.88) for the Netherlands Emergency department Evaluation Database, and AUC of 0.89 (95% CI: 0.89–0.90) in the Danish Multicenter Cohort [18]) and that of previous studies by our group, which observed an AUC of 0.81 (95% CI: 0.75–0.87) [17]. By comparing the ROC curves, we could determine that the IEWS scale’s predictive capacity is significantly superior to that of the NEWS and qSOFA scales, a result similar to that reported in the original study by Candel et al. [18]. Based on this result, we believe that in the emergency department, patients should not only be stratified using scales such as qSOFA and NEWS, but also that the use of the IEWS scale should be routinely considered.

Interestingly, Dadeh AA et al. [24] modified the conventional NEWS scale by adding the lactate value. They demonstrated an AUC of 0.83 for predicting mortality from in-hospital sepsis. However, the AUC value for NEWS without lactate was 0.82, suggesting that the contribution of lactate was not very significant [24]. However, Jo S et al., in a study with 4624 adults, reported a good predictive capacity for mortality with NEWS-L, reaching an AUC of 0.87 (95% CI: 0.85–0.90) [25]. This data is comparable to previous reports from our group with an AUC of 0.84 (95% CI: 0.79–0.90) for NEWSL [17]. Based on these data, we evaluated whether modifying the IEWS and adding the lactate value improved the predictive capacity for death. We were able to document that adding lactate to the original IEWS scale significantly improved the predictive ability of death: IEWS: 0.74 (95% CI: 0.68–0.80); IEWS_L: 0.81 (95% CI: 0.76–0.86); *p* = <0.001. However, the addition of the lactate value to the original score improved the discrimination ability not only of IEWS, but also of NEWS. Based on this, it can be recommended that in addition to performing a risk stratification of patients in the emergency room with suspected sepsis using the IEWS, a serum lactate sample should be obtained to improve the predictive ability and to better classify patients at high risk of death from sepsis who come to the emergency room.

We used the bootstrap method with 1000 repetitions for each test to perform the calibration curves because the data did not have a normal distribution. We determined that the behavior relating to the scale prediction with the observed result was good, especially in the qSOFA and IEWS scales. The NEWS and IEWS_L scales presented some problems at high values. However, we did not consider this slight alteration so significant as to reject the evaluated prediction models.

Regarding the sensitivity found in this study of the scales evaluated, we were able to determine that the NEWS scale has the highest sensitivity with 74%, data very similar to that reported by Wang et al. (73%) [14]. Likewise, the scale with the highest specificity was qSOFA (80%). However, the other scales had acceptable specificity values between 57% and 73%. These data are comparable to those reported in previous studies, such as the meta-analyses of Wang et al. [14] and Qui X et al. [26]. The scale with the highest positive and negative predictive values was IEWS_L, with 41 and 91%, respectively. However, the other scales showed negative predictive values close to 85% and positive predictive values around 35%.

When analyzing the total mortality of the cohort, we found a total mortality rate of 20.6%, a result like that of previous reports with 24.9% [27] and 22.4% [28]. This allows us to apply our analysis results to other populations with similar mortality rates. Additionally, when evaluating mortality due to septic shock, we found a mortality rate of 35.2%. This is comparable to the meta-analysis by Bauer et al. [29], which reported a mortality rate of 34.7% for septic shock, as well as the study by Fleischmann et al., which reported a mortality rate of 26% [30]. Likewise, the mortality rate from septic shock found in our cohort was similar to that reported in other low- to middle-income populations (34.9%) [31].

Interestingly, the sepsis death rate in this study was 10.5%, slightly lower than the 17% mortality rate reported by Fleischman et al. [30]. The association found between having septic shock and in-hospital mortality is worrying, with an odds ratio (OR) of 4.57 (95% CI: 2.70–7.94), which shows the importance of the early recognition of this entity. No differences were found in mortality due to sepsis according to sex, but a significant difference was documented between age and mortality due to sepsis, similarly to what has been previously reported in the literature [1]. Mortality data from studies with elderly patients show that mortality in populations over 70 years of age with sepsis is around 40% [32]. This emphasizes the necessity for a thorough evaluation of older people, considering their increased fragility. It also suggests including age as a prognostic factor in the prediction scales utilized in the emergency department.

### Limitation

The main limitation of this study is that it was conducted in a single hospital, which means that the results should be interpreted cautiously when applying the scores to other populations. However, patient mortality rates were like those of other previously reported populations. The qSOFA, NEWS, IEWS, and IEWS_L scales should not be used to diagnose sepsis. These scores only allow us to determine which patients with suspected sepsis are at higher risk of in-hospital death. The diagnosis of sepsis remains complex, and it is necessary to develop better tools that focus not only on predicting death but also on diagnosing sepsis itself. Our study recommends that physicians and/or nurses in the emergency department triage room use prediction scores to classify the patient population with suspected sepsis and thus offer timely treatment, contributing to improved clinical outcomes.

## 5. Conclusions

The findings of this study indicate that the scale with the best ability to both discriminate and calibrate for predicting in-hospital mortality due to sepsis in the emergency department is the IEWS scale, surpassing both the qSOFA and NEWS scales. Additionally, incorporating serum lactate into this scale significantly enhances its capacity to predict mortality. We recommend conducting further research with the IEWS and IEWS_L scales to facilitate their routine use in the triage area of emergency departments.

## Figures and Tables

**Table 1:** Patient characteristics.

Variable	Survivors (%)	No Survivors (%)	OR	*p*
**Total (n = 383)**	304 (79.4)	79 (20.6)		
Sex(male)	168 (55.3)	43 (54.4)	0.96 (0.58–1.59)	0.995
Age (median (IQ))	72 (57–79)	76 (68–86)		<0.001
Septic shock	101 (33.2)	55 (69.6)	4.57 (2.70–7.94)	<0.001
Glasgow score (median (IQ))	15 (15–15)	15 (14–15)		0.001
Heart rate (mean (sd))	101.16 (23.2)	102.03 (28.8)		0.805
Respiratory rate (median (IQ))	20 (18–22)	22 (19.5–26)		0.028
Oxygen saturation (median (IQ))	92 (88–94)	90 (86–92)		0.015
Systolic pressure (median (IQ))	106 (90–125.2)	98 (70–120)		0.001
Diastolic pressure (median (IQ))	65 (55–75)	59 (41.5–72)		0.009
Temperature (median (IQ))	37.2 (36.5–38.1)	37.1 (36.4–38.4)		0.934
Po2 (median (IQ))	66 (57.3–75.4)	68.5 (58–83.5)		0.07
PaFi (mean (sd))	278.9 (80.3)	225.2 (105.9)		<0.001
Platelet count (median (IQ))	209 (140–335)	243 (143–404)		0.466
Bilirubin (median (IQ))	1 (0.7–1.4)	1 (0.7–1.4)		0.61
Mean art. Pressure (median (IQ))	78.3 (67.2–90.7)	71 (52–89.5)		0.01
Creatinine (median (IQ))	1.2 (0.8–1.6)	1.3 (1.0–1.8)		0.066
Oxygen saturation (median (IQ))	92 (88–94)	90 (86–92)		0.015
Lactate levels (median (IQ))	1.6 (1–2.5)	3 (1.6–5.5)		<0.001
Comorbidities				
Cardiovascular	136 (44.7)	47 (59.5)	1.80 (1.09–3.01)	0.027
Diabetes	76 (25)	20 (25.3)	1.02 (0.56–1.78)	1
Renal insufficiency	22 (7.2)	17 (21.5)	3.50 (1.73–7.00)	<0.001
Immunosuppression	58 (91.1)	12 (15.2)	0.76 (0.37–1.47)	0.526
COPD	24 (7.9)	13 (16.5)	2.30 (1.08–4.72)	0.034
Sepsis origin (%)				0.923
Pulmonary (29.0)	89 (29.3)	22 (27.8)		
Urinal (26.1)	77 (25.3)	23 (29.1)		
Biliary (11.7)	35 (11.5)	10 (12.7)		
Abdominal (6.8)	23 (7.6)	3 (3.8)		
Soft tissues (5.7)	17 (5.6)	5 (6.3)		
Gastroenteritis (5.0)	15 (4.9)	4 (5.1)		
Endocarditis (1.0)	4 (1.3)	0 (0.0)		
Osteomyelitis (0.3)	1 (0.3)	0 (0.0)		
Hospital stay (median (IQ))	9 (6–14.2)	7 (3–13)		0.154
Stay in ICU (median (IQ))	3 (0–6)	4 (2–8)		0.045
qSOFA (median (IQ))	1 (0–1)	1 (1–2)		<0.001
NEWS (median (IQ))	7 (4–9)	9 (7–12.5)		<0.001
IEWS (mean (SD))	11.2 (3.8)	14.8 (3.8)		<0.001
IEWS_L (median (IQ))	13 (10.1–15.9)	19.2 (15.5–22.7)		<0.001

qSOFA, Quick Sequential Organ Failure Assessment; NEWS, the National Early Warning Score; IEWS, the International Early Warning Score; IEWS_L the International Early Warning Score plus the value of arterial lactate; CPOD, Chronic Obstructive Pulmonary Disease; Po2 arterial oxygen pressure; PaFi arterial oxygen pressure divided by the fraction of inspired oxygen.

**Table 2:** Sensitivity, specificity, and predictive values comparing prediction tools.

Score	Threshold	Spec	Sens	NPV	PPV
qSOFA	1.5	0.80	0.46	0.85	0.38
NEWS	7.5	0.57	0.72	0.88	0.30
IEWS	13.5	0.71	0.64	0.88	0.36
IEWS_L	15.6	0.73	0.74	0.91	0.41
NEWS_L	11.05	0.72	0.72	0.90	0.40

SPEC, specificity; SENS, Sensitivity; NPV, negative predictive value; PPV, positive predictive value; qSOFA, Quick Sequential Organ Failure Assessment; NEWS, the National Early Warning Score; IEWS, the International Early Warning Score; IEWS_L, the International Early Warning Score plus the value of arterial lactate; NEWS_L, National Early Warning Score plus the value of arterial lactate.

## Data Availability

The datasets used and/or analyzed during the current study are available from the corresponding author upon reasonable request.

## Abbreviations

NEWS	the National Early Warning Score
COPD	chronic obstructive pulmonary disease
AUC	receiver operating characteristic curve
NPV	negative predictive value
PPV	positive predictive value
IEWS	the International Early Warning Score
